# Ablation of SP-A has a negative impact on the susceptibility of mice to *Klebsiella pneumoniae *infection after ozone exposure: sex differences

**DOI:** 10.1186/1465-9921-9-77

**Published:** 2008-12-04

**Authors:** Anatoly N Mikerov, Rizwanul Haque, Xiaozhuang Gan, Xiaoxuan Guo, David S Phelps, Joanna Floros

**Affiliations:** 1 The Penn State Center for Host defense, Inflammation, and Lung Disease (CHILD) Research, Department of Pediatrics, The Pennsylvania State University College of Medicine, Hershey, PA 17033, USA; 2 Department of Cellular and Molecular Physiology, The Pennsylvania State University College of Medicine, Hershey, PA 17033, USA; 3 Department of Obstetrics and Gynecology, The Pennsylvania State University College of Medicine, Hershey, PA 17033, USA; 4 Department of General Hygiene and Ecology, Saratov State Medical University, Bolshaya Kazachya 112, Saratov 410012, Russia; 5 Capital Institute of Pediatrics, No.2 Yabao Road, Beijing 100020, PR China

## Abstract

**Background:**

Surfactant protein A (SP-A) enhances phagocytosis of bacteria, including *Klebsiella pneumoniae*, by alveolar macrophages. Ozone, a major air pollutant, can cause oxidation of surfactant and may influence lung immune function. Immune function may also be affected by sex-specific mechanisms. We hypothesized that ablation of SP-A has a negative impact on the susceptibility of mice to *Klebsiella pneumoniae *infection after ozone exposure, and that sex differences in the effect of ozone do exist.

**Methods:**

Male and female SP-A (-/-) mice on the C57BL/6J background were exposed to ozone or to filtered air (FA) used as a control and then infected intratracheally with *K. pneumoniae *bacteria. Survival rate was monitored during a 14-day period. In addition, protein oxidation levels and *in vivo *phagocytosis were checked 1 h after inoculation of PBS used as a sham control and after inoculation of *K. pneumoniae *bacteria in PBS, respectively.

**Results:**

We found: 1) ozone exposure followed by *K. pneumoniae *infection decreases survival and alveolar macrophage phagocytic function of SP-A (-/-) mice compared to filtered air exposure (*p *< 0.05), and females are more affected than males; 2) SP-A (-/-) mice (exposed either to ozone or FA) are more susceptible to infection with *K. pneumoniae *than wild type (WT) mice regarding their survival rate and macrophage phagocytic function; the phagocytic function of FA SP-A(-/-) is similar to that of ozone exposed WT. 3) ozone exposure appears to increase infiltration of PMNs, total protein, and SP-A oxidation in WT mice; infiltration of PMNs and total protein oxidation appears to be more pronounced in female mice in response to ozone; 4) ozone exposure increases SP-A oxidation in WT females significantly more than in males.

**Conclusion:**

Absence (i.e. ablation of SP-A in SP-A (-/-) mice) or reduction of functional activity of SP-A (i.e. oxidation of SP-A in WT mice) increases the susceptibility of mice to experimental pneumonia after ozone exposure, and in both cases females are more affected by ozone exposure than males.

## Background

Ozone is one of the major air pollutants. Millions of Americans live in areas with a level of ozone higher than that recommended by the National Ambient Air Quality Standard limit, and this could negatively affect their health. Ozone exposure can cause shortness of breath and coughing, trigger asthma attacks and reduce lung function, often leading to hospital admissions and emergency room visits [1-4]. According to American Lung Association data for 2008, every year almost 400,000 Americans die from lung disease, the number three killer (behind heart disease and cancer), and death rates from lung disease are currently increasing. In 2003, the age-adjusted death rates for females and males were 19.4 and 26.1 per 100,000, respectively. An estimated 651,000 hospital discharges in males and 717,000 discharges in females were attributable to pneumonia in 2005. Lung disease costs the U.S. economy a total of $154 billion. Together, pneumonia and influenza cost the U.S. economy more than $40.2 billion in 2005, according to the American Lung Association.

Pulmonary surfactant plays a key role in lung innate immune defense. Surfactant protein-A (SP-A) is a major surfactant component and innate immune molecule, with activities that include stimulation of chemotaxis of macrophages [5], enhancement of phagocytosis of bacteria [6-10], proliferation of immune cells [11,12], linkage of innate and adaptive immunity [13], and production of proinflammatory cytokines [14-17] by involving, at least in part, NFκB activation [18,19]. Genetically modified mice lacking SP-A are more susceptible to challenge with experimental pneumonia caused by *Pseudomonas aeruginosa *[20], group B *Streptococcus *[21,22], and *Haemophilus influenzae *[22] than WT mice.

As a strong oxidizing agent, ozone can affect SP-A related functions. Ozone-induced oxidation of SP-A reduces its ability to interact with alveolar macrophages [23], inhibits its effect on phosphatidylcholine secretion from alveolar type II cells [24,25], and has a negative impact on its regulation of cytokine production by a human monocytic cell line THP-1 [16,26]. After ozone exposure of SP-A, its aggregation pattern, absorption spectra, gel electrophoretic pattern [25,27], as well as SP-A-mediated lipid aggregation, binding of SP-A to immobilized mannose [24], and the ability of SP-A to enhance phagocytosis of *Herpes simplex *virus and superoxide anion production by alveolar macrophages [23] are all changed. In recent *in vitro *studies we observed that ozone exposure of SP-A decreases its ability to stimulate phagocytosis of both gram-positive and gram-negative bacteria by alveolar macrophages [28]. Taken together, impairment of SP-A activity through oxidation may be one of the mechanisms that contribute to the increased susceptibility to pneumonia.

Sex is one of the known risk factors in lung disease. Males have been shown to suffer more from neonatal respiratory distress syndrome after premature birth when compared to females [29,30]. They are also affected more by idiopathic pulmonary fibrosis and COPD [31]. Young males also have a greater incidence of asthma [31]. On the other hand, females are less afflicted by most types of pneumonia and generally have more favorable outcomes [32,33]. In contrast to lung diseases, in the case of air pollutants, the male disadvantage or female advantage appears to reverse. Elderly females have been shown to have a more pronounced response to ambient air pollution than males [34] and some experimental studies have shown that the lungs of female mice sustain greater damage after naphthalene exposure than those of males [35]. Thus, sex differences in lung health and in response to air pollution may have important biological and clinical consequences.

Alveolar macrophages comprise the first line of lung host defense. Ozone exposure has been associated with impaired functional competence of alveolar macrophages: the release of one of the modulators of inflammation (prostaglandin E2), phagocytosis of particulate immune complexes, superoxide production and production of TNF-α, IL-1, and IL-6, in response to bacterial LPS were all decreased in alveolar macrophages [36]. An ineffective activation of the NF-κB cell signaling pathway was observed when a monocytic cell line, THP-1, was exposed to ozone [37]. Sex may differentially affect alveolar macrophage function and thus contribute to sex differences in pneumonia. Indeed, a decrease in alveolar macrophage phagocytic activity from mice exposed to ozone has been observed [38,39] with female mice affected more than males [39]. Moreover, decreased survival in animals infected with *K. pneumoniae *was demonstrated in response to ozone [39,40] with females being more susceptible than males [39]. However, the mechanisms by which various pollutants influence biological processes are incompletely understood. Furthermore it should be noted that in order to be able to extrapolate findings from rodents exposed to ozone to humans, it is necessary that rodents be exposed to a considerably higher dose of ozone than what would humans are likely to be exposed to. Rodents require exposures to higher ozone concentration than human to reach comparable amounts of ozone in the distal lung [41]. Moreover, resting rodents exposed to 2 ppm showed either comparable PMNs and protein content or lower macrophage number compared to an exercising human exposed to a lower ozone dose (0.44 ppm).

Given that SP-A plays a critical role in assisting phagocytosis in the lung, we hypothesized that either the absence of SP-A expression in the lung of SP-A (-/-) mice or a decrease in SP-A function after oxidation of SP-A in WT mice is one of the factors that contribute to increased susceptibility of mice to pneumonia if exposed to air pollutants, such as ozone. In the present study, we investigated this hypothesis by exposing SP-A (-/-) mice and WT mice to ozone or FA and studying the survival rates, phagocytic activity of alveolar macrophages of SP-A (-/-) mice, and SP-A oxidation in WT mice. The survival and phagocytic activity of alveolar macrophages of WT mice exposed to ozone and infected with *K. pneumoniae *[39] were compared, when appropriate, to those of the SP-A (-/-) mice.

## Methods

### Reagents and media

Tryptic soy agar (TSA) and tryptic soy broth (TSB) were purchased from Sigma (St. Louis, MO) and Dulbecco's PBS was from Invitrogen (Life Technologies, Grand Island, NY). Sterile saline solution (0.9% NaCl) was purchased from Baxter Healthcare Corp. (Deerfield, IL).

### Animals

Pathogen-free male and female C57BL/6 (12 weeks old) WT mice were obtained from Jackson Laboratories (Bar Harbor, ME). The animals were maintained under standard environmental conditions and fed rodent chow and tap water *ad libitum*. The SP-A (-/-) mice on the C57BL/6 background were bred in the Animal Care Facility of Penn State University College of Medicine in isolators under pathogen-free conditions in accordance with approved protocols and policies of Penn State University. Male and female SP-A (-/-) mice were used at the age of 8–12 weeks. The Penn State University Institutional Animal Care and Use Committee approved all procedures involving animals.

### Infection of male and female SP-A (-/-) mice with *K. pneumoniae *bacteria for survival and *in vivo *phagocytosis analyses

Infection with *K. pneumoniae *was performed as we described before [39] with no modifications. In brief, *Klebsiella pneumoniae *bacteria (ATCC 43816) were obtained from the American Type Culture Collection (Rockville, MD) and prepared for infection of mice as described [39]. *K. pneumoniae *bacteria, used in this study, were from the same stock as in our previous work [39]. Moreover, bacteria were taken from the same passage to minimize variation in virulence. The experimental group of mice was exposed to ozone (2 ppm for 3 h), and the control group of mice was exposed to filtered air (FA) at the same time in different chambers. The ozone dose, temperature, flow rate, and humidity levels in chambers were kept at the same levels, as in our previous work with wild type mice [39]. Through all experiments, the incubator temperature was set to room temperature (25°C), the humidity was set to 50%, and the flow rate was 15 L/min through each (FA and ozone) 3.66 L exposure chamber. The ozone dose/duration was chosen in our preliminary work as being optimal for further investigations [42]. Immediately after exposure to ozone, mice were infected intratracheally with *K. pneumoniae *bacteria and allowed to recover from the procedure for inclusion in the study. Animals that died during the procedure were excluded from the analyses.

#### A) Survival study

The *K. pneumoniae *infection dose was ~450 CFU/mouse in 50 μl of PBS. There were 16 independent experiments. Eight experiments involved females and 8 experiments involved males, starting with 10 mice per experiment. Each experiment (10 mice) involved 5 mice exposed to ozone, and 5 mice, exposed to FA. After infection, the mice were monitored for survival twice a day for 14 days. The total number of mice used for analysis in the survival study was 149.

#### B) In vivo phagocytosis study

The *K. pneumoniae *infection dose for assessing *in vivo *phagocytosis was ~1.2 × 10^7 ^CFU/mouse in 50 μl of PBS, and the number of independent experiments was 5 for females and 4 for males, starting with 6 mice per experiment (3 mice were exposed to ozone, and 3 mice to FA). One hour after infecting mice, the lungs were lavaged 3 times with 0.5 ml of 0.9% NaCl and the BAL cells. The alveolar macrophages, were washed similarly and then applied to slides using a cytocentrifuge. The slides were stained with the Hema-3 stain kit (Fisher) and used for phagocytosis analysis with light microscopy. *In vivo *phagocytosis was assessed, as described before for *in vitro *phagocytosis [43]. Mice that died during the procedure were not used for lavage, and BALs with large amounts of red blood cells were not analysed. The total number of mouse samples used for this analysis was 31.

### Analysis of total protein content, SP-A content, oxidized total protein, and oxidized SP-A levels in BAL of male and female WT mice

#### A) Experimental model

WT C57BL/6 male and female mice were used in this study in 4 independent experiments. The experimental design was as described above for *in vivo *phagocytosis with the exception that mice after exposure to FA or to ozone received an intratracheal administration of 50 μl of PBS (sham control), rather than infected with bacterial suspension. One hour after inoculation mouse lungs were lavaged with 0.9% NaCl. The BAL fluid was centrifuged (150 × g, 5 min at 4°C) and the cell pellet was resuspended in 0.9% sodium chloride. Total cell counts were performed using a hemocytometer, and cells were then applied to slides using a cytocentrifuge and stained with the Hema-3 stain kit (Fisher) for differential cell counts. Cell-free supernatants were frozen at -80°C for subsequent protein analyses. The total number of mice used in this study was 39.

#### B) Detection of total protein and SP-A levels in mouse BAL

Total protein concentration in mouse BAL was determined using the Micro BCA Protein Assay (Pierce Biotechnology, IL). SP-A levels in BAL were determined by Western blot analysis. Briefly, 200 μl aliquots of BAL samples were lyophilized and then resuspended with 20 μl of sample buffer. Protein samples were then subjected to SDS-PAGE followed by Western blotting. Blots were incubated with polyclonal rabbit anti-SP-A IgG (1:10,000), and then with secondary antibody (goat anti-rabbit IgG HRP conjugate; 1:25,000) (Bio-Rad). Antibody binding was detected by enhanced chemiluminescence (ECL) and blots were exposed to Kodak X-Omat XAR film (Eastman Kodak Co., Rochester, NY). The film was developed and SP-A levels were quantified by laser densitometry for each BAL sample. The OD × mm^2 ^values were calculated for: SP-A monomer; SP-A dimer; and total SP-A (monomer + dimer).

#### C) Detection of total protein and SP-A oxidation in mouse BAL

Total protein oxidation level was determined using the OxyBlot Oxidized Protein Detection Kit (Intergen, Purchase, NY) as described previously [42] with some modifications. This kit detects carbonyl groups that have been introduced into proteins through oxidation. Briefly, equal volumes (25 μl) of different mouse BAL samples were mixed with 12% SDS at a ratio of 1:1. Samples were then derivatized with 2.5 μl of 10× 2,4-dinitriphenylhydrazine (DNPH) solution and incubated for 10 min at room temperature. Derivatization was stopped and samples were then analyzed by dot blot. Aliquots containing the DNPH-derivatized proteins were brought up to a volume of 500 μl with 100 mM phosphate buffered saline (pH 7.5) and 200 μl of each sample was blotted onto nitrocellulose by vacuum using a 96-well dot-blot apparatus (Bio-Rad) for immunodetection of oxidized proteins, using rabbit anti-DNP and goat anti-rabbit IgG (HRP-conjugated) antibodies. Antibody binding was detected by enhanced chemiluminescence (ECL), and blots were exposed to XAR film (Eastman Kodak Co., Rochester, NY). After this, the protein oxidation levels were quantified by laser densitometry, and OD × mm^2 ^values were calculated for each BAL sample. Oxidized SP-A level was determined by the method of Robinson et al [44] with some modifications. The same samples were used for the detection of SP-A (see above) and oxidized SP-A. Briefly, 200 μl aliquots of BAL samples were lyophilized and then resuspended with 20 μl of sample buffer. Protein samples were then subjected to SDS-PAGE followed by Western blotting. The membrane was washed for 5 minutes in 0.02 M Tris (pH 7.5) with 20% methanol, 5 minute with 2 N hydrochloric acid (HCl), and then treated with 100 μg DNPH/ml 2 N HCl for 5 minutes. The membrane was again washed 3 times with 2 N HCl, 7 times with 100% methanol, and one time with 0.02 M TBS (5 minutes each wash). After, blocking, immunodetection of oxidized SP-A was done as described for the total protein oxidation analysis (see above). The bands in mouse BAL samples, corresponding to human SP-A dimer (used as a marker), were analyzed for the quantitation by laser densitometry and OD × mm^2 ^values were calculated for each mouse SP-A sample.

### Statistics

Survival data were analyzed with log-rank test of Kaplan-Meyer survival curves (cumulative survival, for all 14 days), and with Chi-Square test or Fisher's Exact test (daily survival). Proportions of surviving animals were compared with a Z-test. In all other analyses, a t-test was used. The appropriate analysis is mentioned in the respective figure legends. Data were considered statistically significant when *p *values were less than 0.05.

## Results

### Effect of ozone-exposure on survival of SP-A (-/-) mice after *K. pneumoniae *infection

In this part of the study, we investigated the following hypotheses: i) ozone exposure affects the ability of SP-A (-/-) mice to survive after *K. pneumoniae *infection, and ii) female mice have a higher risk for pneumonia due to infection with *K. pneumoniae *after ozone exposure than males. To address these goals, we first exposed mice to ozone (or to FA which was used as a control) and then infected them with *K. pneumoniae *bacteria. Then, we compared the survival rates of the various groups of mice under study: a) ozone-exposed vs. FA-exposed mice, and b) males vs. females. For this analysis, 149 SP-A (-/-) mice were used. To get further insight into the impact of SP-A on survival, we compared survival of SP-A (-/-) mice with our published data of WT mice [39]. Both the cumulative survival (over the entire 14 day period) as well as daily survival were analyzed.

#### Comparison of survival rates of SP-A (-/-) mice vs. WT mice

**Figure 1:**
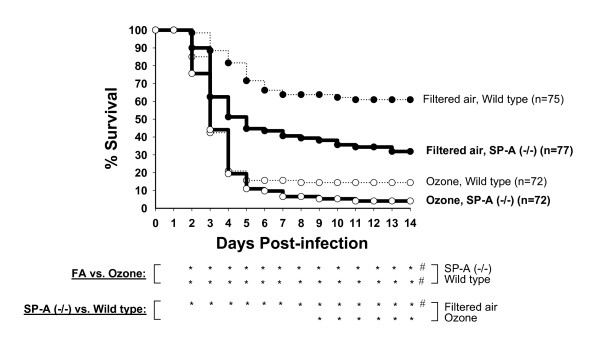
**Effect of ozone exposure on survival rates of SP-A (-/-) mice after *K. pneumoniae *infection: comparison with WT mice**. SP-A (-/-) male and female mice on the C57BL/6 background were used in this study. The experimental design is as described in Methods. Data are represented in percent. FA groups are shown with solid circles and ozone groups – with open circles. Data for WT mice of the same genetic background (shown with dotted lines) are from Mikerov et al. [39], and are shown here for comparison purposes. In this analysis, males and females were analyzed together to study the effect of ozone on survival after pneumonia regardless of sex. Differences were found to be significant if *p *< 0.05 (shown under the Figure). Significant differences by log-rank test (cumulative survival, for all 14 days): ^#^FA group of SP-A (-/-) or WT mice vs. the corresponding ozone SP-A (^-^/-) or WT group; FA or ozone groups of SP-A (-/-) mice vs. the corresponding groups of WT mice. Significant differences by Chi-square test (daily survival): as above for cumulative survival, but for daily analysis each asterisk (*) corresponds to significant differences for the respective day located above.

Data for WT mice [39] were used here to statistically compare with survival rates of SP-A (-/-) mice regardless of sex. Analysis of cumulative survival rates revealed that the survival of FA-exposed SP-A (-/-) mice was significantly lower than that of WT mice (Figure 1). Although the survival of ozone-exposed mice appeared to be lower for SP-A (-/-) mice than for WT mice, these differences were not found to be significant with the log-rank test. When we compared the daily survival rates, we found that the survival rate of FA-exposed SP-A (-/-) mice was significantly lower than that of WT mice on days 2–14 post-infection, whereas the survival rates of ozone-exposed SP-A (-/-) mice was significantly lower than that of WT mice on days 9–14 (see Figure 1). On day 14, the final day of observation, the survival rates (%) were as follows: for SP-A (-/-) mice – 31.9 ± 4.7% and 4.1 ± 2.2% for FA- and ozone-exposed mice, respectively; and for WT mice – 60.9 ± 5.6% and 14.4 ± 3.1% for FA- and ozone-exposed mice, respectively. The daily survival analysis indicates that SP-A (-/-) mice are more susceptible to *K. pneumoniae *infection than WT mice after either FA or ozone exposure.

#### Comparison of survival rates of FA-exposed vs. ozone-exposed SP-A (-/-) mice

**Figure 2:**
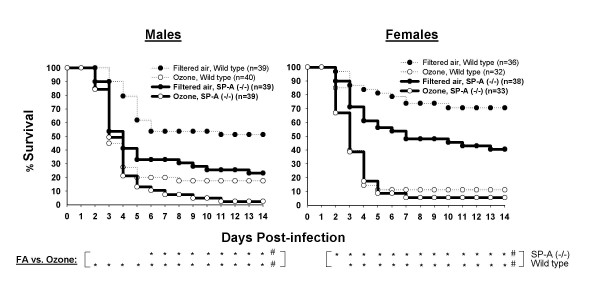
**Sex differences in the effect of ozone exposure on survival rates of SP-A (-/-) mice after *K. pneumoniae *infection**. Experimental design was performed as described in the legend for Figure 1. In this analysis, males and females were analyzed separately to compare the effect of ozone on the survival of infected mice of different sex. Significant differences by log-rank test (cumulative survival, for all 14 days): ^#^FA group of SP-A (-/-) or WT mice vs. the corresponding ozone group. Significant differences by Fisher's exact test (daily survival): as above for cumulative survival, but for daily analysis each asterisk (*) corresponds to significant differences for the respective day located above. Data for WT mice shown by the dotted line are from Mikerov et al. [39].

Analysis of the survival of SP-A (-/-) mice, disregarding sex, demonstrated that ozone exposure significantly decreases both cumulative and daily (days 2–14) survival of mice after pneumonia (Figure 1). Subsequently, we analyzed the effect of ozone exposure on survival rates as a function of sex (Figure 2). Analysis of cumulative survival of SP-A (-/-) mice revealed that the survival rates of male and female mice exposed to ozone are significantly lower than the corresponding rates of those exposed to FA (Figure 2). Daily survival analysis demonstrated significant differences in the survival rates between ozone-exposed and FA-exposed male or female mice on days 6–14 for males and on 2–14 days for females. We concluded that either male or female SP-A (-/-) mice exposed to ozone are significantly more susceptible to *K. pneumoniae *infection than mice exposed to FA. The data [39] from WT mice in Figure 2 demonstrated the same trend, but the survival rates appear to be higher in most cases.

#### Comparison of males vs. females of SP-A (-/-) mice

No significant differences (with either cumulative or daily tests) were observed between survival rates of males and females exposed either to FA or to ozone, a finding similar to that shown previously for WT mice [39]. However, Figure 2 clearly demonstrates that the major differences between survival of male and female SP-A (-/-) mice on day 14 are for the FA-exposed mice (23.1 ± 4.5 and 40.6 ± 7.2 for males and females, respectively), whereas ozone exposure appears to eliminate these differences (2.5 ± 2.5 and 5.6 ± 3.7 for males and females, respectively).

**Figure 3:**
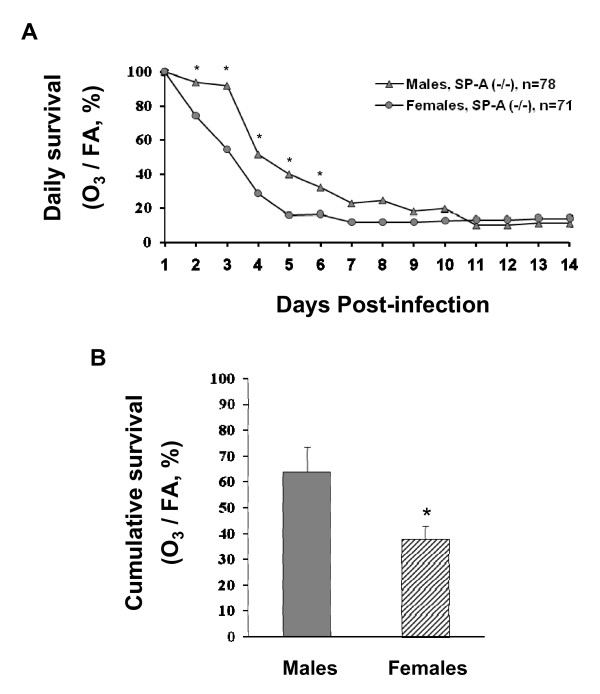
**Comparison of "cumulative" and daily survival rates between males and females of SP-A (-/-) mice after ozone-exposure followed by *K. pneumoniae *infection**. Experimental design was performed as described in the legend for Figure 1 and Methods. Data are presented in percent. 3A: Differences in daily survival between males and females. Survival in the ozone group was calculated as percent of the control group (ozone/FA × 100%). For statistical analysis, proportions (ozone/FA absolute survival rates) for males and females were compared daily with the Z-test for each day. 3B: Differences in the "cumulative" survival between males and females. The experiments from which these data are derived are described in Figure 2 (for SP-A (-/-) mice). The area below the curve was calculated with Sigma Plot 10.0 Software for each experiment. The resulting ratios (ozone/FA × 100%) for males vs. females were compared with a t-test. Significant differences between males and females were noted if *p *< 0.05 (indicated with asterisk, *).

To compare the differential effect of ozone on daily survival of males and females, the influence of both parameters, FA and ozone exposure, was integrated. We calculated the proportions of surviving ozone-exposed mice to surviving FA-exposed mice (control, "normal" conditions) for each day (1–14), and compared these between males and females (Figure 3A). The resulting curves characterize the influence of ozone on the survival rate of mice after pneumonia infection. We observed that females were significantly more susceptible to pneumonia after ozone exposure than males on days 2–6 (Figure 3A). However, after day 6, no differences between males and females were found when ozone/FA (%) values were compared.

To perform a "cumulative" survival analysis, the area (from Figure 2) below the curve (1–14 days) was calculated for ozone-exposed and FA-exposed mice for each experiment. The ozone/FA ratios were then compared between males and females (Figure 3B). This analysis demonstrated that female mice were significantly more susceptible to pneumonia after ozone exposure than males.

We concluded that ozone exposure decreases the survival of female SP-A (-/-) mice after *K. pneumoniae *infection significantly more than it does in males.

### Effect of ozone-exposure on the in vivo (in the lung) phagocytosis of *K. pneumoniae *bacteria by alveolar macrophages isolated from SP-A (-/-) male or female mice

**Figure 4:**
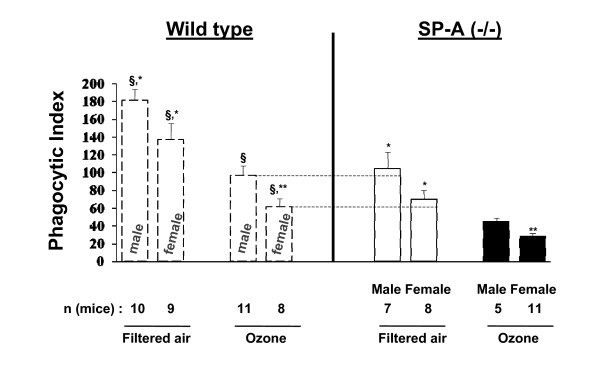
***In vivo *phagocytosis of *K. pneumoniae *bacteria by alveolar macrophages isolated from SP-A (-/-) mice exposed to ozone or FA**. Experimental design is described in Methods. Absolute data were used for this analysis. Data from filtered air and ozone-exposed WT mice (on the left broken lines) are from Mikerov et al. [39] and are presented here for comparison purposes. Reference (dotted lines) in the figure are shown for convenience to demonstrate the similarity of the phagocytic indices of ozone-exposed WT mice and filtered air-exposed SP-A (-/-) mice. Differences were considered significant if *p *< 0.05 with a t-test. *Significant differences between FA exposed and ozone exposed mice of the same sex; **significant differences between males and females after ozone exposure; ^§^significant differences between SP-A (-/-) and WT mice of the same sex and after the same treatment (i.e. ozone-exposed or FA-exposed). The number of independent experiments for female and male SP-A (-/-) mice was 5 and 4, respectively. The number (n) of mice in each group that survived after the procedure is shown.

The phagocytosis level was found to be significantly lower in macrophages from ozone-exposed male or female SP-A (-/-) mice than that from FA-exposed mice (Figure 4). The phagocytic indices of alveolar macrophages isolated from FA-exposed SP-A (-/-) female mice appeared to exhibit lower activity than those from male mice (105.0 ± 18.3 for males and 70.6 ± 9.6 for females) (Figure 4), although the differences did not reach statistical significance (*p *= 0.141). A similar trend was observed previously for alveolar macrophages from FA-exposed WT mice [39]. The phagocytic indices from ozone-exposed SP-A (-/-) mice were found to differ significantly (*p *= 0.006) between males and females (45.3 ± 4.2 for males and 29.4 ± 2.6 for females). The phagocytic index for ozone-exposed females was about 65% of that for males. Thus, the data in Figure 4 show that although ozone exposure causes a significant reduction in the phagocytic index of alveolar macrophages in both sexes, this reduction is greater in females. A similar effect of FA and ozone exposure on the alveolar macrophage phagocytic function has been observed previously for WT mice [39]. We also found that the phagocytic indices of macrophages isolated from SP-A (-/-) mice are significantly lower than those of WT male or female ozone-exposed or FA-exposed mice (Figure 4). Moreover, it was noted that the activities of alveolar macrophages isolated from either male or female WT mice exposed to ozone were comparable with those from FA-exposed SP-A (-/-) mice (Figure 4, each comparison is noted by arrow and dotted line). Data for WT mice are from Mikerov et al [39].

### Analyses of cells, protein, and protein oxidation levels in BAL of male and female WT mice exposed to ozone

To gain insight into mechanisms initiating changes important for the observed sex differences and differences between WT and SP-A (-/-) mice in their survival rates and macrophage phagocytic function, we analyzed BAL from FA- and ozone-exposed WT mice at 1 h time point after inoculation with PBS rather than bacteria. Six types of analyses were performed as described in methods: 1) counting of total cells in whole BAL; 2) differential cell counting of alveolar macrophages/monocytes, lymphocytes, and PMNs on cytospin slides; 3) total protein concentration (μg/ml) in whole BAL; 4) total protein oxidation analysis; 5) SP-A analysis (monomers, dimers, and total (monomers + dimers) SP-A); and 6) oxidized SP-A analysis.

**Figure 5:**
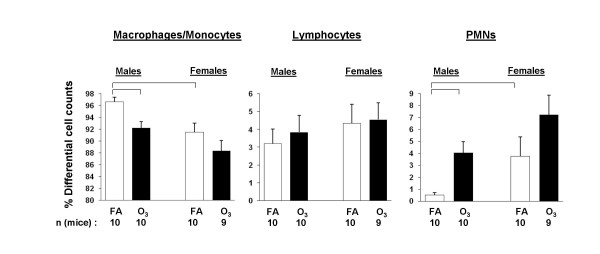
**Differential cell counts in BAL of FA-exposed and ozone-exposed WT male and female mice**. Experimental design was as described for *in vivo *phagocytosis (Figure 4), except that PBS was injected into mice instead of bacteria (see Methods). The percent of polymorphonuclear neutrophils (PMNs), lymphocytes, and macrophages/monocytes was calculated and compared between ozone-exposed and FA-exposed mice and between males and females. The percent of lymphocytes did not differ significantly among different groups. The number of mice in this analysis is shown in the Figure. The number of independent experiments was 4, where each experiment initially involved 6 mice (3 FA-exposed mice and 3 ozone-exposed). Significant differences are shown with lines above the respective bars. Statistical analysis was performed with a t-test and the differences were considered significant if *p *< 0.05.

For the counting of total cells in whole BAL, no significant differences were found between males and females or between ozone-exposed and FA-exposed mouse BALs (data not shown). Differential cell counting revealed an increased number of PMNs in BAL in response to ozone (it was significant only for males), as we have observed before [42]. Moreover, females appeared to be more sensitive to experimental stress than males. They increased the number of PMNs in response to both FA (*p *< 0.05) and ozone (albeit non-significantly) compared to males (Figure 5). The number of macrophages was changed accordingly in the differential cell counts (Figure 5). In addition, the number of PMNs or macrophages from ozone-exposed males was similar to that of FA-exposed females (Figure 5).

**Figure 6:**
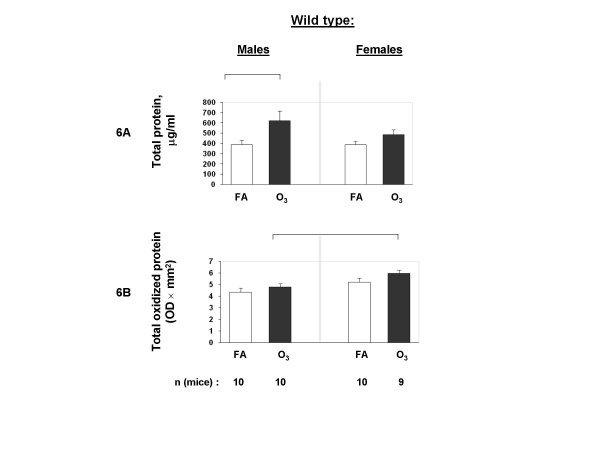
**Total protein oxidation in BAL of FA-exposed and ozone-exposed WT male and female mice**. Experimental design was as described in Figure 5. After the mouse lungs were lavaged, BAL was centrifuged, and the supernatant was used for the estimation of total protein concentration in BAL (6A) and for total oxidized protein analysis (6B) as described in Methods. An aliquot of the same supernatant analyzed in Figure 5 was also used here. The OD × mm^2 ^values were calculated for each sample. FA-exposed groups are shown with opened bars, and ozone-exposed groups – with solid bars. Significance between different groups of mice is shown with connecting lines above the respective bars. Statistical analysis was performed with a t-test and the differences were considered as significant if *p *< 0.05.

**Figure 7:**
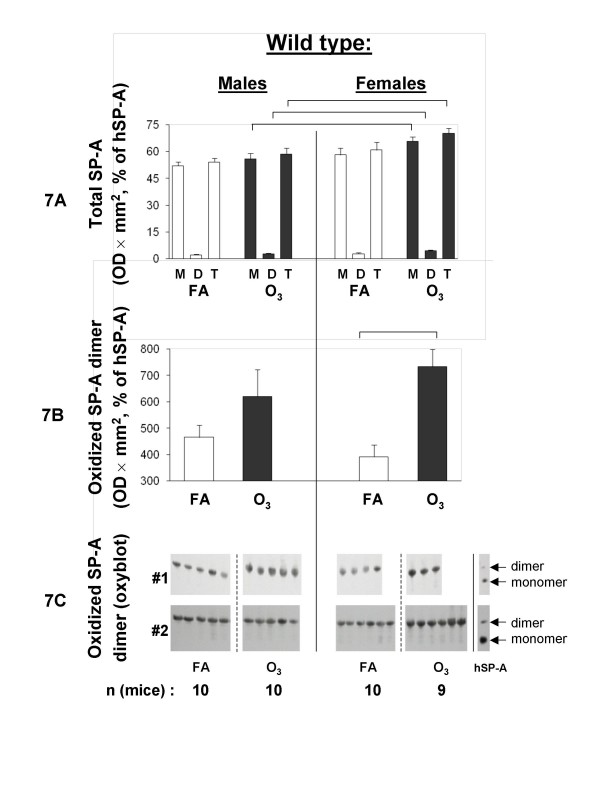
**Oxidized SP-A content in BAL of FA-exposed and ozone-exposed WT male and female mice**. Experimental design was as described in Figure 5. FA-exposed groups are shown with open bars, and ozone-exposed groups – with solid bars. Estimation of total SP-A concentration (7A) and oxidized SP-A dimers (7B) in BAL was performed as described in Methods. Samples used for the estimation of SP-A (7A) and oxidized SP-A (7B) were from same aliquots. For SP-A quantification, SP-A bands corresponding to monomer, dimer, or monomer + dimer (total) SP-A were analyzed. Data for BAL samples in 7A and 7B were from two different blots. To facilitate analysis, data from each mouse BAL sample were normalized to the average of the positive controls (hSP-A) from the two blots, according to the following formula: OD × mm^2 ^of mouse SP-A sample/OD × mm^2 ^of hSP-A times 100%. For oxidized mouse SP-A dimer estimation, oxidized hSP-A dimer was used as a denominator. The oxyblot from the two experiments (# 1 and # 2) depicting SP-A dimer oxidation is shown in 7C. Data were considered to be significant if *p *< 0.05 with a t-test. Significant differences are shown with lines above the respective bars. *M*, monomer SP-A; *D*, dimer SP-A; *T*, total (monomer + dimer) SP-A.

**Figure 8:**
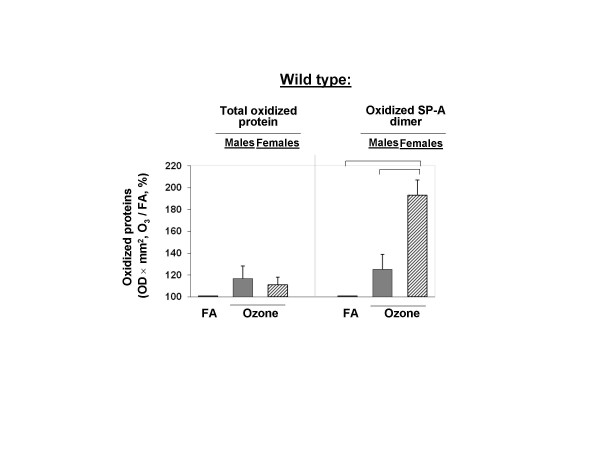
**Net effect of ozone exposure on total protein oxidation and SP-A oxidation in BAL of WT male and female mice**. The data for ozone-exposed mice are from Figures 6B and 7B and they were normalized to the control (FA) that was set equal to 100%. The average of OD × mm^2 ^for FA-exposed mice (control) was calculated for each experiment (n = 4). The net effect of ozone on total protein oxidation and SP-A oxidation in mouse BAL was calculated according to the following formula: OD × mm^2 ^for each ozone-exposed mouse/OD × mm^2 ^of the average for the FA-exposed mice from the same experiment, times 100%. Significant differences are shown with lines above the respective bars. Data were considered as significant if *p *< 0.05 with a t-test.

Total protein concentration was significantly increased in the BAL from males in response to ozone, as we showed before [42], but no significant difference was observed in samples from female mice (Figure 6A). Total protein oxidation appeared to be increased (albeit not significantly) in BAL of ozone-exposed mice with females showing a smaller but significant increase in the levels of oxidized protein in BAL compared to males (Figure 6B). Analysis of the total SP-A (monomer, dimer, and both) demonstrated that SP-A concentration in BAL appeared to increase in response to ozone, and that the values from females were higher than those from males (*p *< 0.05) for all monomer, dimer, and total SP-A for ozone-exposed mice (Figure 7A). Oxidized SP-A dimer analysis (only dimer was found to be oxidized in mouse SP-A) revealed that ozone increased the oxidation of SP-A dimer (with *p *< 0.05 for females only) (Figure 7B and 7C). To assess the net effect of ozone on the total protein oxidation and SP-A oxidation in mouse BAL, the percent of ozone/FA values were calculated. The analysis revealed that ozone appeared to increase oxidation of the total protein in BAL, albeit not significantly. However, ozone specifically increased oxidation of SP-A (*p *< 0.05 for females only) with female mice showing a significantly (*p *< 0.05) higher level of SP-A oxidation than males (Figure 8).

In summary, although the total BAL protein in females appeared to be oxidized significantly more than in males (Figure 6), protein oxidation also appeared to increase in FA-exposed female mice compared to males and thus, as a result the "net effect" of ozone exposure did not show any differences between the two sexes (Figure 8). However, analysis of the proportions of ozone to FA (control) revealed significant differences between females and males in SP-A dimer oxidation (Figure 8). Whether this plays a role in the reduced survival and phagocytic activity of the macrophage from females, remain to be determined.

## Discussion

Surfactant protein A has been shown to play a key role in the innate immune defense of the lung, and ozone, one of the major pollutants in the air, has been shown to affect lung function and surfactant components, including SP-A. Lung immune function may also be affected by sex-specific mechanisms. In this study, we investigated the hypothesis that genetic ablation or functional reduction of SP-A has a negative impact on the susceptibility of mice to *Klebsiella pneumoniae *infection after ozone exposure, and that sex differences on the effect of ozone do exist. For this, we tested survival rate and alveolar macrophage phagocytic function of SP-A (-/-) mice after ozone exposure followed by *K. pneumoniae *infection, as well as the level of total protein and SP-A oxidation in the lung of WT mice after exposure to ozone. We found that: i) ozone exposure significantly decreases survival and alveolar macrophage phagocytic function of SP-A (-/-) mice after intratracheal infection with *K. pneumoniae *bacteria; ii) females are more affected than males, and SP-A (-/-) mice are more affected than WT mice; iii) ozone exposure appears to increase infiltration of PMNs and total protein and SP-A oxidation in WT mice; iv) ozone exposure significantly increases SP-A oxidation in WT females more than in males; and v) infiltration of PMNs and total protein oxidation also appear to be more pronounced in female mice in response to ozone. The results demonstrate that absence (i.e. ablation of SP-A in SP-A (-/-) mice) or reduction of functional activity of SP-A (i.e. oxidation of SP-A in WT mice) increase susceptibility of mice to experimental pneumonia after ozone exposure, and that in both cases females are more affected by ozone exposure than males. Moreover, the findings indicate an important role of SP-A in response to oxidative stress.

Experimental mouse models of infection have been frequently used to study human pneumonia [22,42,45-47] because the molecular mechanisms mediating responses during lung infection in mice and human appear to be more similar than different [48]. Nevertheless caution should be exercised in the interpretation of mouse data to the human situation due to some species differences, including differences in the anatomy, physiology, and the function of the respiratory tract (see ref. [48] for review), the ability of alveolar macrophages to ingest and kill bacteria [49], and potential differences in the inflammatory and/or immune responses in mice and humans [49,50]. However, the advantages of mouse models include the ready access of genetically modified mice where the role of specific molecules can be investigated and thus specific hypotheses can be generated and studied under controlled conditions. In our recent work [43] where rat and human alveolar macrophages were studied for their phagocytic ability (which is relevant to the present study) in the presence or absence of human SP-A1 and SP-A2 variants, similar results were obtained. Moreover, the fact that the observation made in the present study (i.e. females are more susceptible to pneumonia in response to environmentally-induced oxidative stress) is consistent with data from clinical studies, provides further support of the usefulness of the mouse model used in gaining insight into the human situation. In this study, we used genetically-modified mice that do not express the innate host defense molecule SP-A in their lungs to gain insight into the role of SP-A in the susceptibility of male and female mice to experimental pneumonia after oxidative stress caused by ozone.

Data from human clinical studies (except cystic fibrosis patients [51]) typically show that females are less susceptible to pneumonia and have a better outcome [32,33,52-54]. However, in contrast to pneumonia, when the effect of environmental pollutants was analyzed, the male disadvantage or female advantage appears to reverse. Elderly females have been shown to have more pronounced respiratory symptoms in response to ambient air pollution than males [34]. Consistent with the majority of the clinical data and the present study, the lungs of female mice have been shown to sustain greater damage after naphthalene exposure than those of males [35].

Phagocytosis of bacteria *in vitro *has been shown to be assisted by SP-A [6-10], and ozone-induced oxidation of SP-A compromises its ability to enhance phagocytosis [23,28]. *In vivo *findings [42] appear to implicate SP-A in regulating inflammation and limiting epithelial damage in response to ozone. Recent *in vivo *studies showed that ozone exposure decreases both survival and alveolar macrophage phagocytic function in WT mice after *K. pneumoniae *infection [39] with females showing a greater susceptibility in both than males. We hypothesized that a compromise in alveolar macrophage function in response to ozone exposure and *K. pneumoniae *infection may be one of the mechanisms that account for the observed differences in the survival rate between males and females [39]. Specifically, we postulated that SP-A plays an important role in the overall activity of the alveolar macrophage. In the present study, we investigated the role of SP-A in survival rates and the *in vivo *alveolar macrophage phagocytic function of *K. pneumoniae*-infected SP-A (-/-) mice in response to ozone-induced oxidative stress. The observations made revealed the same trend as in WT mice [39], although the SP-A (-/-) mice had lower survival rates for both FA- and ozone-exposed mice. However, the phagocytic indices of alveolar macrophages from ozone-exposed WT mice were at levels similar to those of FA-exposed SP-A (-/-) mice for each sex. Moreover, ozone exposure of SP-A (-/-) mice reduced the activity of their alveolar macrophages even further, indicating that the alveolar macrophages are functionally hypoactive compared to alveolar macrophages derived from WT mice. These observations together support the notion that alveolar macrophages from SP-A (-/-) mice function sub-optimally [42,46,55] compared to those from WT mice.

Several possibilities may account for this, including the following: 1) SP-A may "prime" the alveolar macrophages (reviewed in [56]) to be ready to respond in the face of a challenge (i.e. infection, oxidative stress, other). The priming may involve expression of cell surface proteins including ICAM-1 and CD14 [57] and Toll-like receptors (our unpublished observations, [58]). These cell surface molecules may then recognize and transduce various signals, whether from microorganisms [59,60] or toxic agents [61] such as ozone. When alveolar macrophages or macrophage-like cell lines are incubated in the presence of SP-A, NF-κB activation is enhanced [18,62], cytokine production is increased [15,57,63] and there may be modulation of expression or function of other effector molecules. Thus, in the absence of SP-A, as in the case of SP-A (-/-) mice, the function of the alveolar macrophage is likely to be compromised. The function of alveolar macrophages is expected, under the proposed scenario, to also be compromised in the presence of SP-A modifications, whether these are due to oxidation [28,39], nitration [64,65], or proteolytic damage [66]. An *in vivo *study also provided some support for this [39]. 2) SP-A was shown previously to be oxidized immediately after the ozone-induced oxidative stress and before increases in total protein oxidation were observed [42]. A similar trend was observed in the present study, although this reached significance for female mice. This observation points to the possibility that SP-A plays an antioxidant role as previously suggested [67-69]. The antioxidant role of SP-A in WT mice may involve its ability to scavenge reactive oxygen species resulting from ozone exposure and thus protect other molecules from immediate oxidative damage. In the absence of SP-A (as in SP-A (-/-) mice) there may be an increased basal level of oxidative stress (as if an external agent such as ozone exposure was present) and this may compromise the function of the alveolar macrophages. Observations from the present study (i.e. alveolar macrophages from ozone-exposed WT mice and FA-exposed SP-A (-/-) mice exhibit similar phagocytic activity) are consistent with this possibility. 3) Oxidation of SP-A immediately after ozone exposure may have an important role in signaling that relates to the presence of reactive oxygen and nitrogen species [70-72]. Oxidation of SP-A was determined by assessing carbonylation, an irreversible oxidative modification. However, it is possible that SP-A is also subject to reversible oxidative modifications involving cysteine thiol side chains that may be physiologically important [73] in signaling of redox-active molecules. Cysteine residues in SP-A have been shown to be oxidized following ozone exposure [74], although the specific nature of this modification has not been identified. Whether SP-A is subject to reversible oxidative modifications of its cysteine thiol groups and whether sex differences exist in these putative modifications, remain to be determined.

The number of PMNs in WT male mice is significantly increased in response to ozone-induced oxidative influences [42]. However, we observed a differential effect of ozone on the number of neutrophils in BAL of male and female mice following *K. pneumoniae *infection (Figure 6). Female mice appeared to have more PMNs in their BAL after ozone-exposure than males, although statistical significance was not achieved. Moreover, FA-exposed (control) female mice also had more (*p *< 0.05) PMNs in their BAL than males, although the number of PMNs was similar between males and females when BALs from untreated (not subject to FA or ozone exposure) males and females were compared (data not shown). This may be a consequence of a markedly increased rate of air exchange in FA-exposed mice versus untreated mice. The above information indicates that female mice may be more sensitive to stress, because even under control conditions, with room air in the airflow the number of PMN cells was increased compared to that in males. It is important to note, that recruited PMNs maintain the capacity to generate proinflammatory cytokines and reactive oxygen species, and this may further contribute to the acute lung injury [75]. Moreover, exposure to low levels of ambient ozone has been shown to lead to defective neutrophil phagocytosis and intracellular killing in humans [76]. Thus, the increased PMNs in females may explain, in part, their increased susceptibility. We speculate that, as assessed by the increased recruitment of PMNs, females exhibit a higher sensitivity to stress and, in particular to ozone-induced oxidative stress, and that this negatively impacts the survival and the phagocytic activity of the macrophage in *K. pneumoniae *infection model.

No significant differences were found in the levels of total protein oxidation between FA and ozone-exposed mice for either males or females. However, oxidation of dimeric SP-A was observed in both males and females at the one hour post-exposure time point. These levels achieved significance for females, and although males showed the same trend, significance was not reached, even though in our previous study with WT male mice [42] the differences in levels of SP-A oxidation between FA and ozone exposures was significant. The reasons for this apparent discrepancy are not clear. The relative content of SP-A dimer is small compared to total SP-A content. However, under normal conditions most of the SP-A in the lung is associated with lipid and only a relatively small amount of SP-A is free of lipid [77,78] and presumably available for non-surfactant related functions. It is possible that this "free" SP-A is reflected here by the SP-A dimer that is subject to oxidative modification and this modification, as discussed above, may be physiologically important in signaling, in the redox state of the lung, modulation of macrophage activity, or other.

In summary, absence or functional impairment of SP-A may be one of the mechanisms that contribute to the increased risk of hospitalization for pneumonia when ambient ozone levels are high [4,79,80]. The present study indicates that sex differences exist with regards to survival and phagocytic activity of the alveolar macrophage in the *K. pneumoniae *infection model in response to ozone-induced oxidative stress with females being more at risk; this is consistent with most relevant clinical observations. Moreover, the present study indicates a role for SP-A in oxidative stress and macrophage function. We speculate that a compromise in innate immunity may add a further burden, especially in the female lung, to its ability to cope with *K. pneumoniae *infection in the face of environmental oxidant-producing stressors. Future studies that take sex into account are warranted.

## Conclusion

Absence (i.e. ablation of SP-A in SP-A (-/-) mice) or reduction of functional activity of SP-A (i.e. oxidation of SP-A in WT mice) increases the susceptibility of mice to experimental pneumonia after ozone exposure, and in both cases females are more affected by ozone exposure than males.

## Competing interests

The authors declare that they have no competing interests.

## Authors' contributions

ANM set up the infection model, the *in vivo *phagocytosis model, planned and carried out all experiments, analyzed and interpreted data, and wrote the manuscript. RH participated in the analyses of cells, protein, and protein oxidation levels in BAL of male and female WT mice exposed to ozone. XGan participated in experiments of survival study. XGuo participated in SP-A (-/-) mouse breeding and handling. DSP contributed to the planning of experiments, interpretation of data, and writing of the manuscript. JF contributed to the design of the project, analysis and interpretation of data, and the writing of the manuscript. All authors read and approved the final manuscript.
